# Transcriptomic Analysis of Glycolysis-Related Genes Reveals an Independent Signature of Bladder Carcinoma

**DOI:** 10.3389/fgene.2020.566918

**Published:** 2020-12-23

**Authors:** Zezhong Mou, Chen Yang, Zheyu Zhang, Siqi Wu, Chenyang Xu, Zhang Cheng, Xiyu Dai, Xinan Chen, Yuxi Ou, Haowen Jiang

**Affiliations:** ^1^Department of Urology, Huashan Hospital, Fudan University, Shanghai, China; ^2^Fudan Institute of Urology, Huashan Hospital, Fudan University, Shanghai, China; ^3^National Clinical Research Center for Aging and Medicine, Fudan University, Shanghai, China

**Keywords:** glycolysis, signature, prognosis, survival analysis, bladder carcinoma

## Abstract

**Background:**

Bladder carcinoma (BC) is one of the most prevalent and malignant tumors. Multiple gene signatures based on BC metabolism, especially regarding glycolysis, remain unclear. Thus, we developed a glycolysis-related gene signature to be used for BC prognosis prediction.

**Methods:**

Transcriptomic and clinical data were divided into a training set and a validation set after they were downloaded and analyzed from the Cancer Genome Atlas (TCGA) and Gene Expression Omnibus (GEO) databases. Gene-set enrichment analysis (GSEA) and differential analysis were used to screen differentially expressed genes (DEGs), while univariate Cox regression and lasso-penalized Cox regression were employed for signature establishment. To evaluate the prognostic power of the signature, receiver operating characteristic (ROC) curve and Kaplan–Meier (KM) survival analysis were also used. Additionally, we developed a nomogram to predict patients’ survival chances using the identified prognostic gene signature. Further, gene mutation and protein expression, as well as the independence of signature genes, were also analyzed. Finally, we also performed qPCR and western blot to detect the expression and potential pathways of signature genes in BC samples.

**Results:**

Ten genes were selected for signature construction among 71 DEGs, including nine risk genes and one protection gene. KM survival analysis revealed that the high-risk group had poor survival and the low-risk group had increased survival. ROC curve analysis and the nomogram validated the accurate prediction of survival using a gene signature composed of 10 glycolysis-related genes. Western blot and qPCR analysis demonstrated that the expression trend of signature genes was basically consistent with previous results. These 10 glycolysis-related genes were independent and suitable for a signature.

**Conclusion:**

Our current study indicated that we successfully built and validated a novel 10-gene glycolysis-related signature for BC prognosis.

## Introduction

Bladder carcinoma (BC), a highly aggressive malignant lesion, accounted for more than one million new diagnoses and approximately 200,000 eventual deaths in 2018 (Bray et al., 2018). Two determinative risk factors affecting the survival of patients with BC are tumor relapse and metastasis (Nadal and Bellmunt, 2019). Thus, to improve BC patients’ survival and recurrence, it is necessary to conduct studies focusing on early detection and the prevention of the growth and spread of BC.

Glycolysis is a major and crucial component of metabolism whose malignant changes represent carcinogenesis (Hanahan and Weinberg, 2011). In normal cells, glucose is metabolized through the tricarboxylic acid (TCA) cycle, during which the metabolic intermediate pyruvic acid is oxidized into carbon dioxide, water, and releases adenosine triphosphate (ATP) to provide energy (Koppenol et al., 2011). In cancer cells, even when oxygen is sufficiently present, a large quantity of lactate is generated through glycolysis with a reduction of ATP (Vaupel and Mayer, 2012), which is known as the “Warburg effect” (Biswas et al., 2012).

Metabolic changes in cancer cells consume large quantities of glucose and produce massive amounts of lactate, and hence contribute to the growth, invasion, and metastasis of tumors (Mayer and Vaupel, 2013). Therefore, glycolysis is an important contributor to BC incidence and progression (Woolbright et al., 2018a). Previous studies have indicated that the Warburg effect plays a central role in cancer invasion and metastasis (Lebelo et al., 2019). Multiple studies on mechanisms of glycolysis in BC have demonstrated that the increase of the glycolytic rate in cancer cells is associated with the overexpression of several core glycolysis-related genes, such as glucose transporter type 1, lactic dehydrogenase A, and hexokinase 1/2 (Miao et al., 2013; Massari et al., 2016; Yu et al., 2017; Garcia et al., 2019). Additionally, there are several other key metabolic pathways involved in the regulation of the Warburg effect, including the phosphoinositide 3-kinase (PI3K)/protein kinase B (AKT)/mammalian target of rapamycin (mTOR) pathway (Mossmann et al., 2018; Reyes-Farias and Carrasco-Pozo, 2019). Several novel BC tumor biomarkers have been detected over the past several decades with the progression of molecular biology and bioinformatics. In spite of this development, major BC risk signatures have been designed on the basis of genes related to multiple phenotypes. There is no study concerning the specific analysis of glycolysis-related prognostic biomarkers to date (Wang et al., 2018). Thus, in this study, we focused on the transcriptomic analysis and clinical characteristics of glycolysis-related genes.

We first employed gene-set enrichment analysis (GSEA) to determine glycolysis-related pathways in BC and then screened the associated differentially expressed genes (DEGs). Gene signature establishment and validation was completed using the Cancer Genome Atlas (TCGA) and Gene Expression Omnibus (GEO) databases, after which, resulting genes were analyzed from the perspective of protein expression and gene mutation. Finally, a personalized nomogram was constructed for clinical analysis and decision-making. Thus, this study provides a novel view for investigations in BC.

## Materials and Methods

### Data Sources and Processing

Transcriptome data of BC, including 56,753 genes from 19 normal samples to 411 BC samples were obtained from TCGA database. Subsequently, we extracted clinical information, such as ID, age, stage, T, N, overall survival (OS), and survival state to establish a gene signature and analyze prognostic survival. The following data were excluded if: (1) no OS time was recorded; and (2) clinical characteristics were incomplete; for example, if OS time was less than 30 days. We then acquired BC GSE13507 gene expression and clinical information as the validation set, which included 24,323 genes from 10 normal tissues to 165 tumor groups by applying the same selection and exclusion methods described above. Finally, sample profiles and gene expression were combined for further analysis.

### Glycolysis-Related Differential Gene Selection

Bladder carcinoma samples in TCGA were first investigated using GSEA analysis. We divided transcriptomic genes into normal and tumor sample groups as the expression dataset. Next, gene sets related to glycolysis were obtained from the Molecular Signatures database v7.1 (MSigDB): BIOCARTA_ GLYCOLYSIS_PATHWAY, GO_GLYCOLYTIC_PROCESS, HA LLMARK_GLYCOLYSIS, KEGG_GLYCOLYSIS_GLUCONEOG ENESIS, and REACTOME_GLYCOLYSIS. Enrichment analysis was performed according to the default weighted enrichment statistics, and the analysis was randomly repeated 1,000 times. According to the GSEA analysis, genes with statistical significance (*p* < 0.05, normalized enrichment score (NES) > 1, and false discovery rate (FDR) < 0.25) were screened as glycolysis-related genes in BC. Afterward, we performed differential analysis on 19 pairs of normal and tumor samples using the R package “limma,” genes were adopted as significant if |log FC| > 1, *p* < 0.05.

### Construction of a Glycolysis-Related Gene Signature

Univariate Cox regression analysis was used to identify prognosis-related genes from glycolysis-related DEGs; genes were statistically significant if the hazard ratio (HR) ≠ 1, *p* < 0.05.

After choosing 393 BC samples from TCGA as a training set and 165 samples from GSE13507 as a validation set, with the aim to narrow the scope of the screen, we further filtered the remaining genes using lasso-penalized Cox regression analysis with the R package “glmnet.” Thus, we acquired the optimal value with minimum deviation using lasso regression with a 10-fold cross-validation and built a 10-gene predictive signature. Following the calculation of the risk score value, a risk score formula was established as follows:

$$\mathrm{Risk}\mathrm{score}=\sum_{i=1}^{n}\beta_{i}*{\text{Exp}}_{i}$$

Based on the median value of the risk score, the training set was separated into high- and low-risk groups. Similarly, we used the original signature containing the same genes to calculate the respective median risk score value in the validation set and accomplished a similar division of the group.

### Signature Validation in the Training and Validation Sets

We verified the high- and low-risk groups categorized by risk score value through survival tests in the training and validation sets, using the R packages “survminer” and “survival”. Moreover, a single gene’s Kaplan–Meier analysis was also determined to test its independence.

We accomplished a prognostic analysis of clinicopathological information in the training and validation sets, plotting groups based on age, stage, T, and N (age: ≤65, >65; stage: stage I-II and stage III-IV; T: T1-2 and T3-4; and N: N0 and N1-3). For the capacity evaluation of 393 samples with complete clinical information in TCGA, univariate and multivariate Cox regression analysis was used to identify independent prognostic factors, including age, stage, T, and N; *p* < 0.05 was regarded as significantly different. Furthermore, after establishing the receiver operating characteristic (ROC) curve in the training and validation sets, respectively, the area under the curve (AUC) value and the surface of the area under the ROC curve was calculated to estimate the predictive accuracy: the larger the AUC value, the more accurate the prediction.

### Construction and Validation of a Predictive Nomogram

In this study, a nomogram was used to assign points for signature contributors, such as age, stage, and risk score, depending on their contribution to the outcome variable. We assigned the independent prognostic factors referred to above to construct a nomogram by using R package “rms.” The maxscale was set at 100. By summing separate points, we could obtain a total score to assess individual prediction. Each variable on the corresponding line was marked with a scale that represented the value range, while the length of the line reflected the factor’s contribution. Discrimination validation was then performed by calculating the Harrell’s concordance index (C-index) using “survConcordance” in the “survival” R package. The C-index has been widely used to calculate the discrimination between the predictive value of a COX regression signature and the real value in survival analysis. The resulting C-index value ranged from 0.7 to 0.9 and was considered to be highly accurate. To compare the prediction and actual outcomes, we constructed a calibration curve of 1-, 3-, and 5-year OS that was capable of evaluating the fit between the signature and the real situation using the R package “rms.”

### External Validation of the Signature

To further validate the expression of signature genes from the angle of protein expression, we retrieved immunohistochemistry (IHC) staining images of prognosis-related genes in normal and BC tissues from the Human Protein Atlas (HPA) online database^1^.

At the same time, we tested four gene mutation forms “Missense Mutation,” “Truncating Mutation,” “Amplification” and “Deep Deletion” in cBioPortal^2^ to explore possible gene mutation forms in the signature. Then, we tested the correlation value of the signature genes in the search tool for the retrieval of interacting genes/proteins (STRING) database^3^, limiting the screening threshold value to the minimum required interaction score: medium confidence (0.400); the line thickness indicated the strength of data support. Additionally, the R package “corrplot” was also used to calculate the correlation of related genes: the lower the correlation value between genes, the higher the independence between genes, and a better clinical significance could be demonstrated.

### Functional Enrichment Analysis of Correlated Genes

We first explored 50 most relevant genes which interactive with glycolysis-signature by STRING database. Then, gene ontology (GO) and Kyoto Encyclopedia of Genes and Genomes database (KEGG) enrichment analysis were performed to reveal the relevant biological functions and pathways of these correlated genes. GO and KEGG analysis were applied and visualized by “clusterProfiler” and “enrichplot” package in R (version 4.0.2). *P*-value < 0.05 were considered as enriched and statistically significant.

### RNA Extraction and qRT-PCR Analysis

Total RNA was extracted from BC tissues using TRIzol reagent (Invitrogen) following the manufacturer’s protocol. BC tissues were obtained by the patients at Huashan hospital, Fudan University (Shanghai, China). All the participants provided Informed consent and our research was approved by the Ethics Committee. Then RNA was reverse transcribed into cDNA with the cDNA Reverse Transcription Kit (Takara Biotechnology). The following qRT-PCR was performed as we previously described (Chen et al., 2020). Primer sequences are listed in Supplementary Material.

### Western Blot Analysis

Western blotting was carried out according to standard protocols (Ma et al., 2019; Yang et al., 2020). Antibodies used in the western blot are listed as follows: anti-PAM (Abcam, ab237488), anti-SLC16A3 (Abcam, ab234728), anti-Stanniocalcin 1 (Abcam, ab124891), anti-CLDN9 (Proteintech, 16196-1-AP), anti-HDAC4 (Proteintech, 17449-1-AP), anti-KDELR3 (Proteintech, 27632-1-AP), anti-Versican (Abcam, ab19345), anti-triosephosphate isomerase (Abcam, ab170894), anti-CASP6 (Abcam, ab108335), anti-CHST6 (Abcam, ab154332), anti-N-Cadherin (Abcam, ab76011), anti-Vimentin (Abcam, ab92547), anti-PARP1 (Abcam, ab32138), anti-GAPDH (Proteintech, 60004-1-Ig), anti-Phospho-AKT (Proteintech, 66444-1-Ig), anti-p62 (Proteintech, 66184-1-Ig), Phospho-mTOR (Cell Signaling Technology, #5536), and anti-p-S6 Kinase (Cell Signaling Technology, #9209).

## Results

### The Glycolysis Process and Related Genes Were Active in BC

**FIGURE 1:**
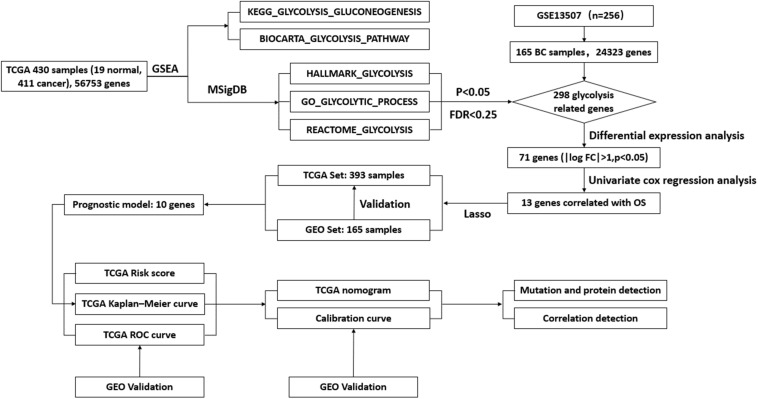
Flow chart of the key points during the process.

**FIGURE 2:**
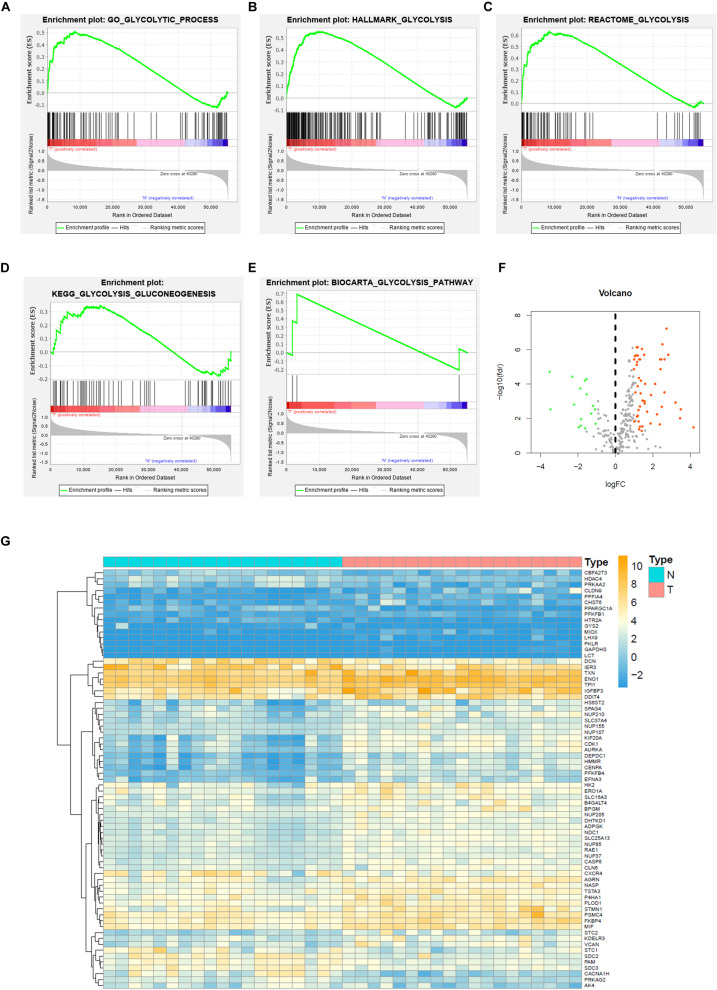
Gene set enrichment analysis (GSEA) and glycolysis-related DEGs of BC in TCGA. **(A)** GO_GLYCOLYSIS_PROCESS, **(B)** HALLMARK_GLYCOLYSIS, **(C)** REACTOME_GLYCOLYSIS, **(D)** KEGG_GLYCOLYSIS_GLUCONEOGENESIS, **(E)** BIOCARTA_GLYCOLYSIS_PATHWAY, and **(F)** Volcano plot of DEGs for TCGA dataset, and **(G)** Heatmap of GEGs for TCGA dataset.

To describe the basic process and thinking of this study, a flow chart with key points was developed (Figure 1). In an attempt to determine whether glycolysis was activated in BC, we searched glycolysis-related pathways in the MSigDB database and analyzed the activation of five pathways using GSEA, of which the GO_GLYCOLYTIC_PROCESS, HALLMARK_GLYCOLYSIS, and REACTOME_GLYCOLYSIS pathways provided results with a NES = 1.50 and FDR *q*-val = 0.030 (Figure 2A); NES = 1.74 and FDR *q*-val < 0.0001 (Figure 2B); NES = 1.70 and FDR *q*-val = 0.014 (Figure 2C), separately; while outcomes from the BIOCARTA_GLYCOLYSIS_PATHWAY and the KEGG_GLYCOLYSIS_GLUCONEOGENESIS pathway had an NES = 1.07 and FDR *q*-val = 0.435 (Figure 2D), and NES = 1.00 and FDR *q*-val = 0.459 (Figure 2E). Correspondingly, we selected 378 genes with statistical significance from the former three pathways as potential glycolysis-related genes. We extracted 19 pairs of matched cancer and para-cancer samples from TCGA database for differential analysis (|log FC| > 1, *p* < 0.05). By doing this extraction, 16 genes with low expression and 55 genes with high expression were obtained (Figures 2F,G). DEG expression was extracted for further analysis as well.

### Construction and Survival Analysis of a Glycolysis Signature

**FIGURE 3:**
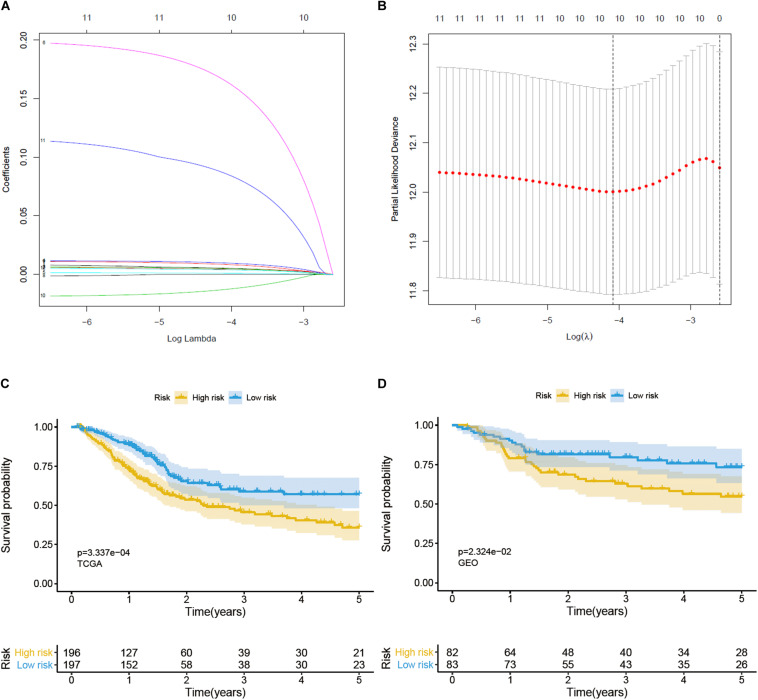
Construction and verification of a prognostic gene signature for BC patients. **(A)** Lasso coefficient profiles of 12 prognostic genes. **(B)** Lasso-penalized Cox regression with ten-fold cross-validation obtained 10 prognostic genes by using minimum lambda value. **(C)** Survival analysis in training set. **(D)** Survival analysis in validation set.

Univariate Cox regression analysis was used to identify prognosis-related genes from glycolysis-related DEGs; a gene was regarded as significantly different if HR ≠ 1, *p* < 0.05 (Table 1). We then performed lasso-penalized Cox regression, using TCGA as the training set and GEO as the validation set, and finally constructed a predictive signature, including 9 risk genes and 1 protective gene (Figures 3A,B). Next, a risk score was calculated, employing the formula here: peptidyl-glycine alpha-amidating monooxygenase (PAM) expression ^∗^0.005734 + solute carrier family 16 member 3 (SLC16A3) expression ^∗^0.004647 + stanniocalcin-1 (STC1) expression ^∗^0.005327 + triosephosphate isomerase (TPI1) expression ^∗^0.001106 + histone deacetylase 4 (HDAC4) expression ^∗^0.176777 + KDEL endoplasmic reticulum protein retention receptor 3 (KDELR3) expression ^∗^0.009246 + carbohydrate (*N*-acetylglucosamine 6-O) sulfotransferase 6 (CHST6) expression ^∗^0.093539 + versican (VCAN) expression ^∗^0.004233 + caspase-6 (CASP6) expression ^∗^(−0.014811). Patients were then divided into high- and low-risk groups on the basis of the median value. Patients in the high-risk group performed worse than those in the low-risk group in both the training (*p* < 0.05) and validation (*p* < 0.05) sets, according to OS analysis (Figures 3C,D). Consistent with our conclusion, the prognosis analysis curve of each gene also indicated that the protective gene CASP6 had a better prognosis when highly expressed, while risk genes had a worse prognosis when highly expressed (Supplementary Figure 1).

**TABLE 1:** Summary of the differential analysis, univariate cox analysis, and Coef. of the genes in BLCA.

Gene	Differential analysis		Univariate Cox	Coef.
	LogFC	*P*-value	*P*-value	
PAM	−1.081	0.001	0.005	0.005734
SLC16A3	1.245	0.008	0.012	0.004647
STC1	−1.956	0.018	0.043	0.005327
CLDN9	3.491	0.001	0.024	0.010418
TPI1	1.007	9.13e-06	0.025	0.001106
HDAC4	−1.856	3.06E-05	0.012	0.176777
KDELR3	1.556	2.47E-04	0.017	0.009246
CASP6	1.080	5.79E-05	0.037	−0.014811
CHST6	2.371	0.012	0.018	0.093539
VCAN	1.707	0.001	0.018	0.004233
GYS2	−3.464	0.001	0.022	
DCN	−2.293	4.31E-06	0.029	
PLOD1	1.140	6.55E-07	0.024	

**FIGURE 4:**
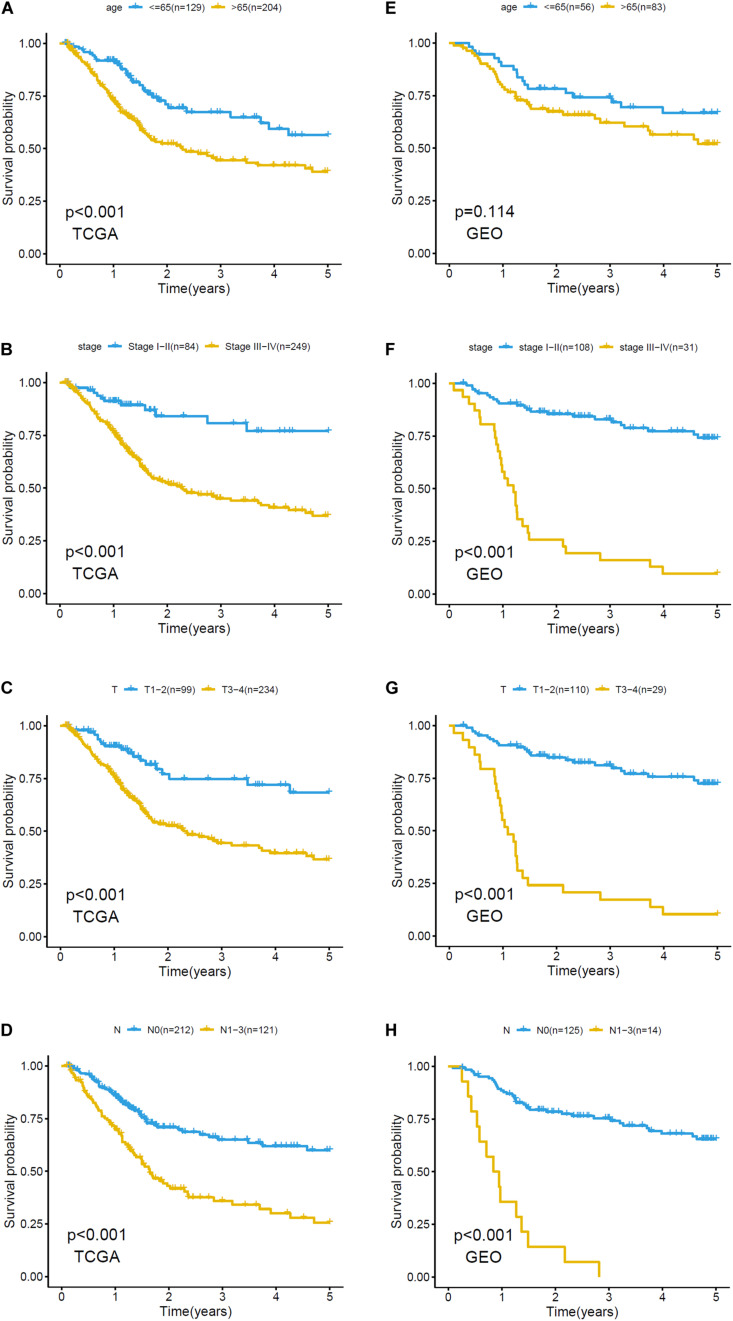
Kaplan–Meier plots of clinicopathological analysis in training set and validation set. Survival analysis of training set based on **(A)** age: ≤65, >65, **(B)** stage: stage I-II and stage III-IV, **(C)** T: T1-2, T3-4, **(D)** N: N0, N1-3. Survival analysis of validation set based on **(E)** age: ≤65, >65, **(F)** stage: stage I-II and stage III-IV, **(G)** T: T1-2, T3-4, and **(H)** N: N0, N1-3.

The relationship between clinicopathological grouping and prognosis in the training and validation sets was analyzed, respectively. Patients with older age, higher pathological stage, or higher level of T and N in the training set had a worse prognosis (Figures 4A–D); the results (except age) in the validation set were similar (Figures 4E–H).

### Validation of Signature Grouping

**FIGURE 5:**
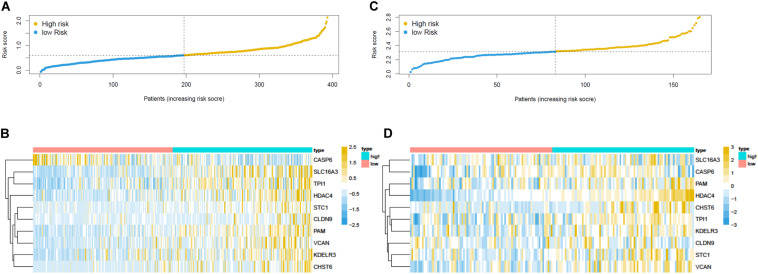
Prognostic risk score model analysis of training and validation set. **(A)** The risk score analysis, **(B)** heatmap in the training set. The expression levels were scaled by *z*-score on single gene level. **(C)** The risk score analysis and **(D)** heatmap in the validation set.

**FIGURE 6:**
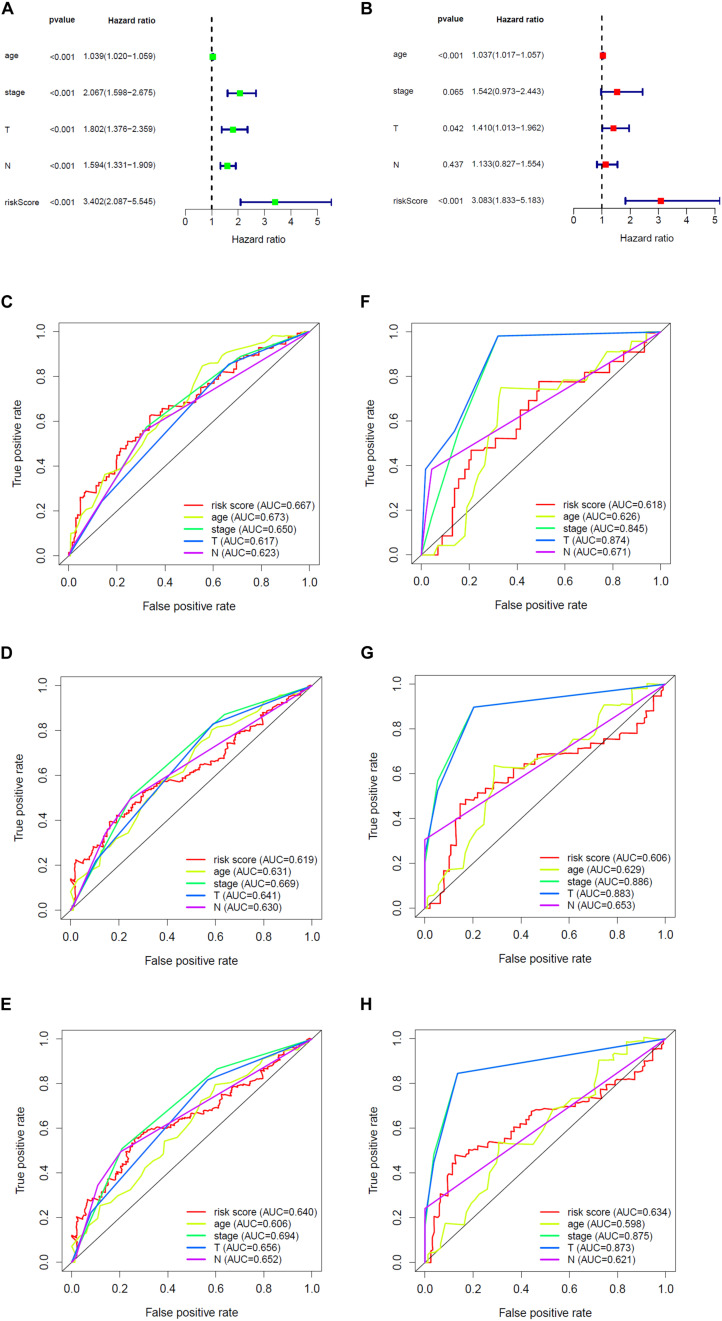
The prognostic effect analysis in the TCGA dataset and ROC curves in the training and validation set. **(A)** Univariate Cox regression analysis of BLCA in training set. **(B)** Multivariate Cox regression analysis of BLCA in training set. The ROC analysis of training set for survival prediction by glycolysis-related gene signature at **(C)** 1-year, **(D)** 3-year, and **(E)** 5-year. The ROC analysis of validation set for survival prediction by glycolysis-related gene signature at **(F)** 1-year, **(G)** 3-year, and **(H)** 5-year.

Overall survival status, survival time, and gene expression were further explored to evaluate the group division of the signature. Patients were ranked from left to right according to the risk score that was calculated in the training and validation sets (Figures 5A,C). The heatmaps demonstrated the differential expression of glycolysis genes in high-risk and low-risk groups in the training (Figure 5B) and validation (Figure 5D) sets. High- and low-risk groups were clearly divided with the aid of the risk score. The prognostic value of the risk score was detected using univariate and multivariate analysis in TCGA-Urothelial Bladder Carcinoma (BLCA). Age (HR = 1.039, 95% CI = 1.020–1.059, *p* < 0.001), stage (HR = 2.067, 95% CI = 1.598–2.675, *p* < 0.001), T (HR = 1.802, 95% CI = 1.376–2.359, *p* < 0.001), N (HR = 1.594, 95% CI = 1.331–1.909, *p* < 0.001), and risk score (HR = 3.402, 95% CI = 2.087–5.545, *p* < 0.001) were significantly associated with OS in the univariate analysis (Figure 6A). Multivariate analysis revealed that age (HR = 1.037, 95% CI = 1.017–1.057, *p* < 0.001) and risk score (HR = 3.083, 95% CI = 1.833–5.183, *p* < 0.001) were also significantly associated with OS, and also indicated that the risk score was an independent prognostic predictor (Figure 6B).

We then built a time-dependent ROC curve to estimate the predictive accuracy. The AUC value and the surface of the area under the ROC curve were also calculated. In TCGA, the AUC for 1-, 3-, and 5-year OS was 0.667, 0.619, and 0.640, respectively (Figures 6C–E), while in GEO, it was 0.618, 0.606, and 0.634 (Figures 6F–H), which indicated that the signature was capable of discriminating to some extent.

### Establishment and Validation of a Nomogram

**FIGURE 7:**
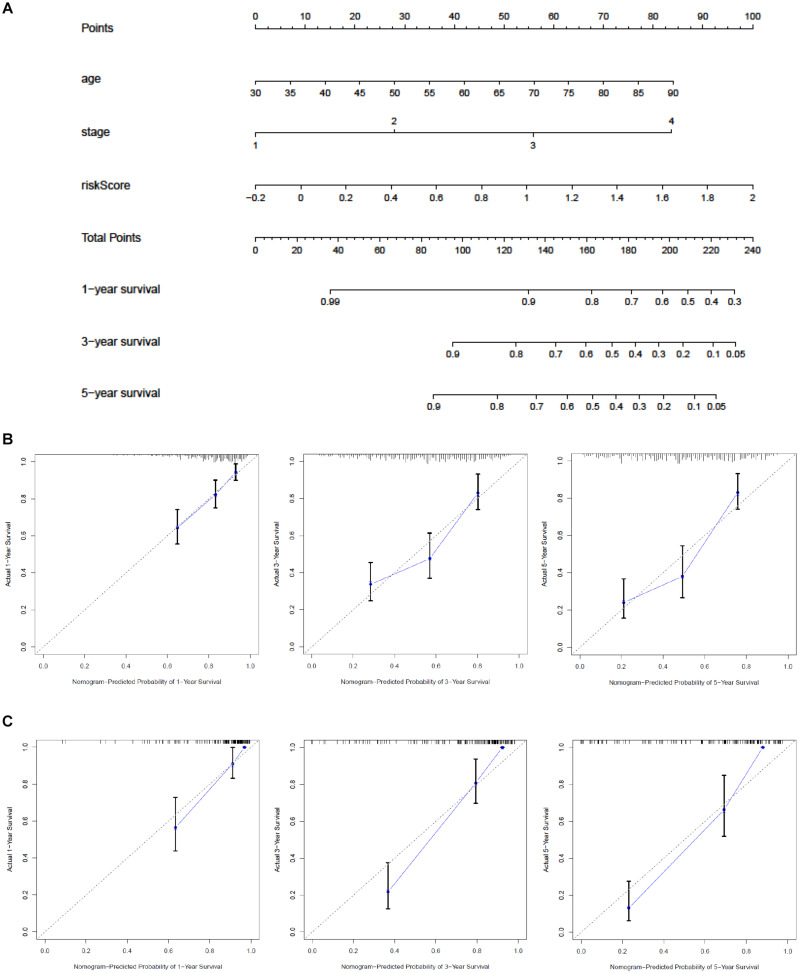
Construction of a nomogram based on the glycolysis-related signature. **(A)** Nomogram combining the signature with stage and risk score. Calibration curve of the nomogram at 1, 3, and 5 years **(B)** in the training set **(C)** and in the validation set. The *Y*-axis stands for actual survival while *X*-axis represents the nomogram-predicted probability of 1-/3-/5-year survival.

A nomogram was built to predict the 1-, 3-, and 5-year OS in TCGA and to promote the creation of a clinically applicable method that may predict BC patients’ survival. With three prognostic factors, age, stage, and risk score included, we built the gene signature combining clinicopathological features with risk scores to predict survival chances of BC patients (Figure 7A). The C-index values of the nomogram in TCGA and GEO were 0.71 and 0.851. The diagonal line in the calibration curve symbolized the most ideal result; the closer it was to the diagonal line, the more consistent it was with the actual situation. Thus, we found that the 1-, 3-, and 5-year OS predicted by our nomogram fit the real situation through the calibration curve, which demonstrated the nomogram’s reliability (Figures 7B,C).

### External Validation of the Gene Signature Through Mutation and Protein Expression

**FIGURE 8:**
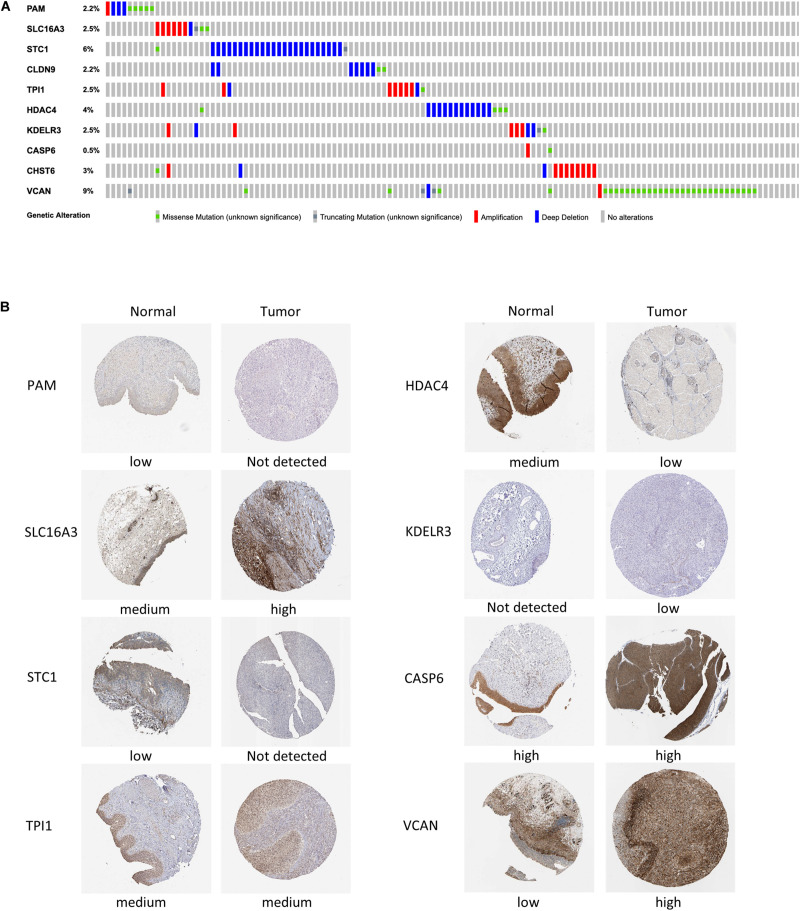
Mutation analysis and protein expression of the genes in the signature. **(A)** Mutations of 10 genes in BC patients. **(B)** Immunohistochemistry staining of the proteins in BC tissues and paired normal tissues based on The Human Protein Atlas.

Mutations in four out of the 10 genes in the signature were analyzed in the cBioPortal database, indicating that the VCAN mutation rate (9%) ranked the highest, and its main mutation type was missense mutation. The mutation rate of CASP6 (only 0.5%) was the lowest (Figure 8A).

We then collected the protein expression data of eight genes in BC and normal samples from the HPA database for validation (Figure 8B), which revealed that the differences in the protein expression were in accordance with those in the gene expression. Nevertheless, the immunohistochemical images of Claudin 9 (CLDN9) and CHST6 were not included, so their expression level could not be shown.

### Independence and Functional Analysis of Signature Genes

**FIGURE 9:**
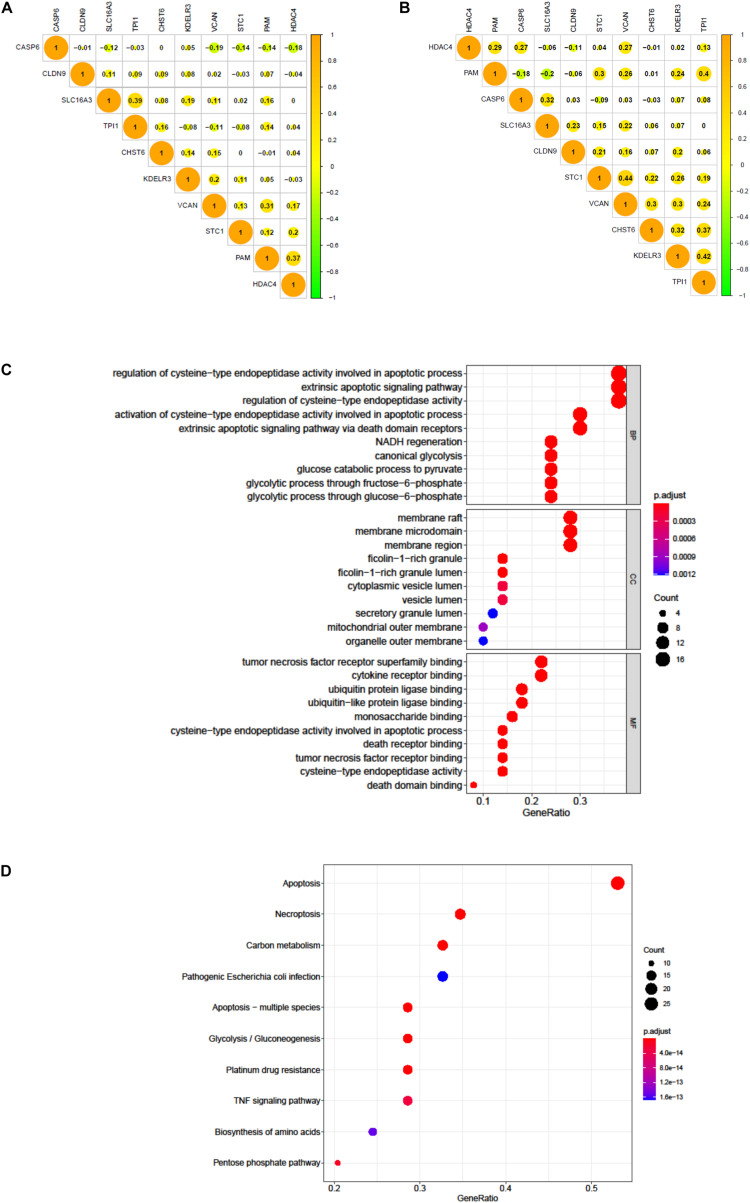
The correlation of 10 genes in BC patients. **(A)** The correlation of 10 genes in 411 BC patients of TCGA dataset. **(B)** The correlation of 10 genes in 165 BC patients of GEO dataset. **(C)** GO analysis of 50 most correlating genes, *p* < 0.05. **(D)** KEGG analysis of the 50 most correlating genes, *p* < 0.05.

We tested the correlation and constructed a PPI network of all glycolytic-related DEGs through the STRING database (Supplementary Figure 2). The results confirmed that our 10 signature genes have low possibility of interaction with each other. Meanwhile, the expression correlation analysis of these 10 genes in TCGA and GEO also demonstrated their independence (Figures 9A,B). Furthermore, we also excavated 50 genes that were most interactive with these 10 genes. We next applied GO and KEGG functional enrichment analysis to reveal potential biological process that these 50 genes may participate in Figures 9C,D. KEGG results specifically indicated that apoptosis, necroptosis, and platinum drug resistance were remarkably enriched in spite of glycolysis.

### Validation of the Signature in Local Samples

**FIGURE 10:**
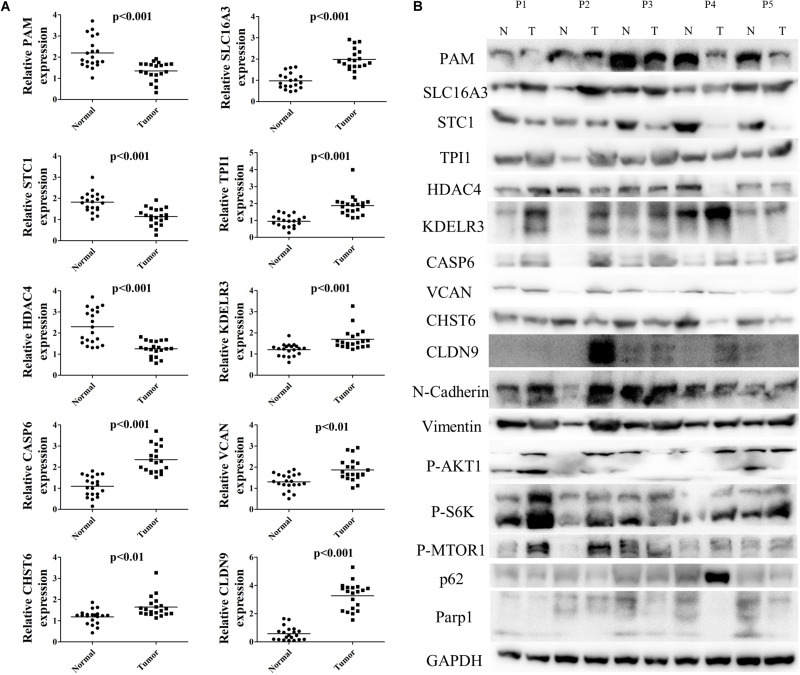
Validation of mRNA and protein levels of 10 hub genes in signature. **(A)** The mRNA expression of 10 signature genes in 20 pairs BC tissues and para-carcinoma tissues. **(B)** The protein expression level of 10 signature genes and relevant pathway markers in five pairs BC tissues and para-carcinoma tissues.

To further validate differential mRNA expression of the 10 hub genes in BC tissue samples, qRT-PCR analysis was conducted in 20 paired carcinoma and para-carcinoma samples (Figure 10A). Relative PAM, STC1, and HDAC4 expression were down-regulated, whereas CASP6, CHST6, SLC16A3, TPI1, KDELR3, VCAN, and CLDN9 were highly expressed in carcinoma tissues compared to the para-carcinoma tissues. The mRNA expression levels were completely consistent with previous analyses. Hereafter, we performed western blot analysis to further investigate protein expression levels of signature genes. As Figure 10B shows, protein expression levels were approximately coherent with mRNA expression levels. Nevertheless, some of the gene expression such as CLDN9, CHST6 were not completely matched with previous findings, which may be largely explained by post-transcriptional regulation. Additionally, we also tested several markers of relevant pathways altered by glycolysis (Figure 10B). Results demonstrated that vimentin, P-akt, and P-s6k were up-regulated in BC tissues, while expression of N-Cadherin, P-mTOR, p62, and PARP1 were decreased in tumor. Taken together, all these results confirmed that signature hub genes are differentially expressed and could effectively revealed tumor progression and prognosis.

## Discussion

Metabolic changes play an important role in BC incidence and growth (Massari et al., 2016). As a key part of metabolism, glycolysis is considered to be influential for BC (Ritterson Lew et al., 2015). Existing studies on glycolysis in BC are mainly confined to the regulation between molecules and pathways, in particular, the core genes, such as those for the glucose transporter family and the pyruvate kinase family (Huang et al., 2018; Woolbright et al., 2018b). While studies on other molecular targets related to survival prognosis remain insufficient, a gene signature that can be used to predict prognostic risk at the level of gene expression, mutation, and prognostic values of glycolysis-related genes was constructed.

Our results suggested that the prognosis signature established on the basis of glycolysis-related genes effectively distinguished patients’ prognostic survival and outlined individualized prediction, according to clinical characteristics. We first verified the significance of glycolysis in BC. Then, we extracted related genes to facilitate and innovate the filtration, as previous studies only detected potential downstream pathways by analysis of the GSEA. Then, we selected two data sets, including three major races’ samples worldwide from TCGA and GSE13570 for analysis and validation, indicating that our conclusions were comprehensive. Furthermore, other studies of glycolysis-related signatures, such as those of hepatocellular carcinoma (Jiang et al., 2019) and lung adenocarcinoma (Zhang L. et al., 2019), are mainly built based on the data from TCGA without external verification. Therefore, our results were more reliable. In addition, we also applied qPCR and western blot to detect the hub genes’ mRNA and protein expression levels in our local samples. As glycolysis is a hallmark of tumor progression, we also detected several biological markers’ expression in our tissues. As our previous expected, epithelial-mesenchymal transition (EMT), metabolic processes were both increased, while apoptosis was obviously inhibited in tumor samples. All results above indicated that our signature could effetely predict patients’ prognosis.

Unfortunately, limited pairs of cancer and para-cancer samples from TCGA were selected for differential analysis because there were in total 411 BC cases included in the TCGA data set and only 19 para-cancer samples were recorded. Thus, we decided not to simply conduct differential analysis with all samples involved, or a statistical offset would occur. Instead, we selected a total of 10 genes, which were reported to be capable of regulating glycolytic function or tumor growth, as signature genes through differential expression screening, univariate Cox regression, and lasso-penalized Cox regression. VCAN, a member of the versican proteoglycan family, is involved in cell adhesion, proliferation, and migration (Zhang Y. et al., 2019c). VCAN can also be used for BC survival prediction, as one of the microenvironment-related prognostic genes when applying its cell adhesion function (Luo et al., 2019). Studies have shown that VCAN also acts as a core protein in the EMT, as its upregulation promotes leukemia cell invasion (Yang et al., 2019). Trisphosphate isomerase 1 (TPI1) is a glycolysis enzyme that is described as a glycolysis-relevant biomarker of pancreatic ductal adenocarcinoma by several researchers; its increase in expression is positively associated with an adverse reaction to chemotherapy (Follia et al., 2019). Stanniocalcin-1 (STC1) is a secreted glycoprotein whose increasing expression is linked to poor prognosis, progression, and metastasis of a variety of tumors, such as breast cancer (Chang et al., 2015), ovarian cancer (Zhang C. et al., 2019), and colon cancer (Pena et al., 2013). HDAC4 plays a critical role in transcriptional regulation, cell cycle progression, and developmental events; overexpressed HDAC4 is associated with the clinical progression and prognostic deterioration of esophagus carcinoma; moreover, it positively regulates the EMT, and hence contributes to tumor growth (Zeng et al., 2016). PAM, CLDN9, CHST6, SLC16A3, KDELR3, and CASP6 are regarded as biomarkers or prognosis genes in the signature, with reports revealing their expression in the areas of the prostate (Jimenez et al., 2001), endometrial lung adenocarcinoma (Wang Z.H. et al., 2019), Uveal Melanoma (Li et al., 2018), and gastric cancer (Wang Z. et al., 2019).

Of course, there are some limitations to the study. First, the prediction of the signature, whether in the training or validation sets, was relatively unstable due to lack of samples, which may result in a deviation. We have combined our efforts to establish our own database for further validation and exploration. Second, although the TCGA database is currently the most comprehensive and complete database in the cancer field, there are numerous missing clinical information on BC: for example, there were many truncated data in OS and excessive Mx category samples occupied the M stage. Therefore, to solve this problem, we estimated the 5-year survival and only employed clinical data of the T and N stages.

## Conclusion

We established a signature of 10 glycolysis-related genes that effectively distinguished high- and low-risk patients from a novel perspective, and verified that the correlation and interaction between the 10 genes was weak, which demonstrated that, to a certain extent, the genes of our signature were representative.

## Data Availability Statement

The datasets presented in this study can be found in online repositories. The names of the repository/repositories and accession number(s) can be found in the article/Supplementary Material.

## Ethics Statement

The studies involving human participants were reviewed and approved by the Huashan Hospital, Fudan University (Shanghai, China). The patients/participants provided their written informed consent to participate in this study.

## Author Contributions

ZM and HJ designed the study. CY, ZZ, and SW performed the assays. CX conducted the statistical analyses. XD and ZC performed the western blot. XC and YO applied qPCR analysis. CY and ZM wrote the manuscript. All authors read and approved the final manuscript.

## Conflict of Interest

The authors declare that the research was conducted in the absence of any commercial or financial relationships that could be construed as a potential conflict of interest.

## Footnotes

1 http://www.proteinatlas.org

2 http://cbioportal.org

3 https://string-db.org
