# Supplementary material for: Transcriptomic Analysis of Glycolysis-Related Genes Reveals an Independent Signature of Bladder Carcinoma
Source: Front Genet. 2020 Dec 23;11:566918. doi: 10.3389/fgene.2020.566918 (PMC7786194; doi:10.3389/fgene.2020.566918)
Supplement: Supplementary file 1 [file Image_1.PDF]

Supplementary Material

1.1 Supplementary Figure

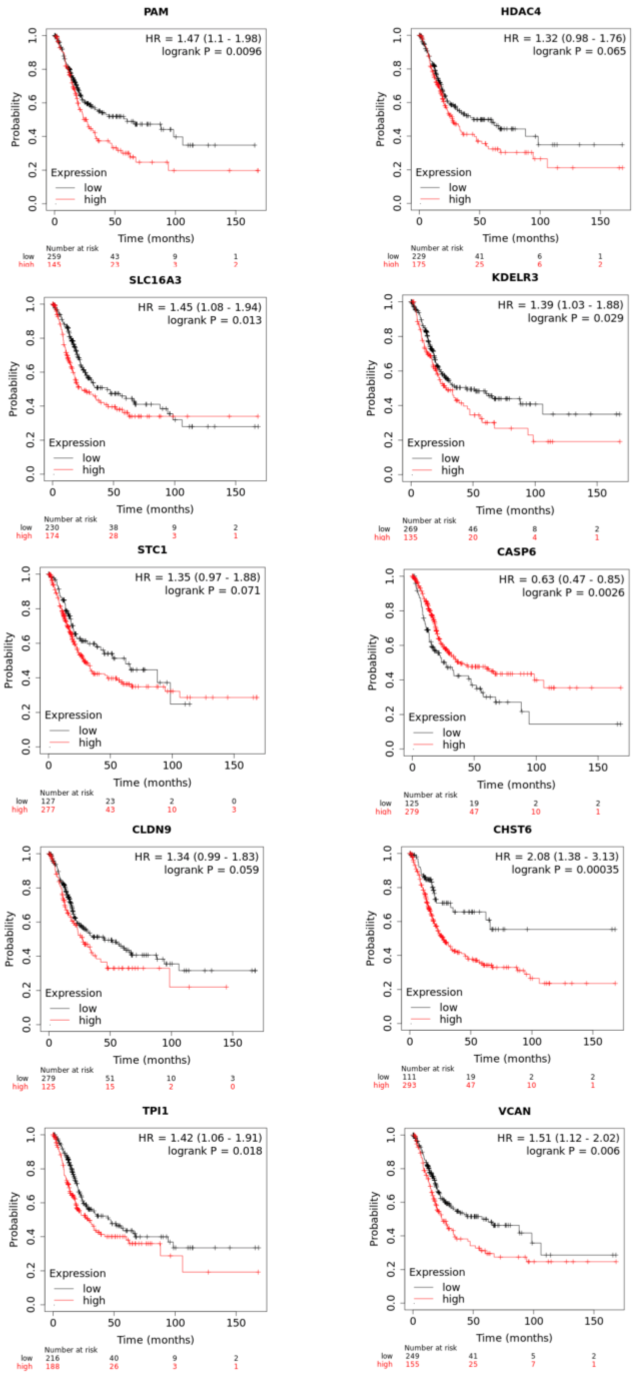

Supplementary Figure 1

Supplementary Figure 1. Kaplan–Meier survival analysis of PAM, HDAC4, SLC16A3, KDELR3, STC1, CASP6, CLDN9, CHST6, TPI1, VCAN.

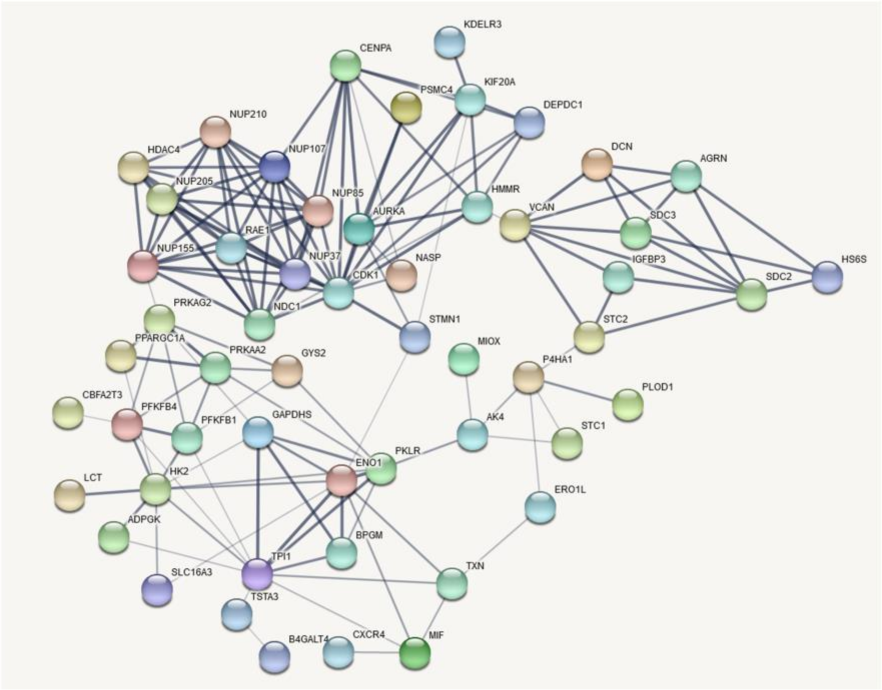

Supplementary Figure 2

Supplementary Figure 2. The network of 71 DEGs downloaded from STRING database.
