# Supplementary material for: Transcriptomic Analysis of Glycolysis-Related Genes Reveals an Independent Signature of Bladder Carcinoma
Source: Front Genet. 2020 Dec 23;11:566918. doi: 10.3389/fgene.2020.566918 (PMC7786194; doi:10.3389/fgene.2020.566918)
Supplement: Supplementary file 2 [file Table_1.DOCX]

Supplementary Material

# Supplementary Data

Primer sequences of qRT-PCR are listed as follow:

| Gene | Forward | Reverse |
| --- | --- | --- |
| CASP6 | ATGGCGAAGGCAATCACATTT | GTGCTGGTTTCCCCGACAT |
| CHST6 | GTTTGATGCCTATCTGCCTTGG | ACGATGCGTAGGTTGAGCG |
| CLDN9 | CGGCTGCACTGCTTATGCT | GAGGGGATGGAGTAGCCCA |
| HDAC4 | GGCCCACCGGAATCTGAAC | GAACTCTGGTCAAGGGAACTG |
| KDELR3 | TCCCAGTCATTGGCCTTTCC | CCAGTTAGCCAGGTAGAGTGC |
| PAM | AGGAGCCGAAAAATGAGCGG | GGAGCTGCAATCAAGTAACCC |
| SLC16A3 | CCATGCTCTACGGGACAGG | GCTTGCTGAAGTAGCGGTT |
| STC1 | GTGGCGGCTCAAAACTCAG | GTGGAGCACCTCCGAATGG |
| TPI1 | CTCATCGGCACTCTGAACG | GCGAAGTCGATATAGGCAGTAGG |
| VCAN | GTAACCCATGCGCTACATAAAGT | GGCAAAGTAGGCATCGTTGAAA |
